# Distribution of Root-Associated Bacterial Communities Along a Salt-Marsh Primary Succession

**DOI:** 10.3389/fpls.2015.01188

**Published:** 2016-01-05

**Authors:** Miao Wang, Pu Yang, Joana Falcão Salles

**Affiliations:** ^1^Research Group of Microbial Community Ecology, Groningen Institute for Evolutionary Life Sciences, University of GroningenGroningen, Netherlands; ^2^Research Group of Microbial Ecology, Groningen Institute for Evolutionary Life Sciences, University of GroningenGroningen, Netherlands

**Keywords:** root-associated bacteria, salt marshchronosequence, primary succession, plant selective force, soil type

## Abstract

Proper quantification of the relative influence of soil and plant host on the root-associated microbiome can only be achieved by studying its distribution along an environmental gradient. Here, we used an undisturbed salt marsh chronosequence to study the bacterial communities associated with the soil, rhizosphere and the root endopshere of *Limonium vulgare* using 454-pyrosequencing. We hypothesize that the selective force exerted by plants rather than soil would regulate the dynamics of the root-associated bacterial assembly along the chronosequence. Our results showed that the soil and rhizosphere bacterial communities were phylogenetically more diverse than those in the endosphere. Moreover, the diversity of the rhizosphere microbiome followed the increased complexity of the abiotic and biotic factors during succession while remaining constant in the other microbiomes. Multivariate analyses showed that the rhizosphere and soil-associated communities clustered by successional stages, whereas the endosphere communities were dispersed. Interestingly, the endosphere microbiome showed higher turnover, while the bulk and rhizosphere soil microbiomes became more similar at the end of the succession. Overall, we showed that soil characteristics exerted an overriding influence on the rhizosphere microbiome, although plant effect led to a clear diversity pattern along the succession. Conversely, the endosphere microbiome was barely affected by any of the environmental measurements and very distinct from other communities.

## Introduction

Assessments of microbial diversity have revealed soils—the habitats where plants and microbes live together and build highly diverse interactions—as being among the most biologically diverse on Earth (Curtis et al., 2002; Gans et al., 2005; Philippot et al., 2013; Saleem and Moe, 2014). From the perspective of plants, soil represents a reservoir of microbes that can potentially affect their biomass, fitness and stress tolerance (Saleem et al., 2007; Buée et al., 2009; Faure et al., 2009; Lambers et al., 2009; Lugtenberg and Kamilova, 2009; Chaparro et al., 2012). From the perspective of microorganisms, plant roots represent true microbial oases, by creating a very selective environment with lower biodiversity but higher activity compared with the bulk soil (Kuzyakov, 2002; Berendsen et al., 2012; Cibichakravarthy et al., 2012), the so-called rhizosphere effect (Smalla et al., 2006; Hartmann et al., 2008; Faure et al., 2009).

The rhizosphere community also represents the source of endophytic bacteria, which cross the root barrier and colonize the plant tissues, the endosphere (Sessitsch et al., 2002; Compant et al., 2005; Hardoim et al., 2008). The selective force plants exert on the endophytic communities, therefore, is predicted to be stronger than that on the rhizosphere. Endophytes differ according to their root colonization mechanisms. Some soil-inhabiting bacteria might become endophytic by stochasticity (e.g., via colonization of natural wounds), being considered as passenger endophytes (Hardoim et al., 2008). Rare rhizosphere microbiome selected stochasticly by different *Nicotiana* root was found to contribute major root colonists (Saleem et al., 2015). Opportunistic and competent endophytes, however, show particular root colonization characteristics (e.g., chemotaxis response), in addition, competent endophytes could be well adapted to the plant environment and lead to the beneficial maintenance of the plant-microbe association (Hardoim et al., 2008). Moreover, endophytes are better buffered against abiotic stresses, common in the complex soil environment (Hallmann et al., 1997). Studies have indicated that endophytic bacterial communities are dynamic over time, with endophytes following the development of plant growth (Hardoim et al., 2008; van Overbeek and van Elsas, 2008). Overall, from the bulk soil and rhizosphere microbiome, plant roots recruit a very rare fraction of microbiota as endophytes, whereas their distribution across root morphological gradient may vary depending on plant and soil types (Saleem et al., 2015).

As discussed above, it is evident that plants exert a very strong selective force on the microbiome associated with the roots (rhizosphere and endophytes). However, this selective force is rather different at different locations, which has been justified by differences in agricultural practices (Salles et al., 2006), sampling sites (Costa et al., 2006), or soil type (Inceoǧlu et al., 2010). This is not surprising, considering that soil properties such as pH or organic carbon, known as major drivers of soil bacterial community assembly (Fierer and Jackson, 2006; Lauber et al., 2009; Dini-Andreote et al., 2015), vary according to soil type (Marschner et al., 2001) or land use (Lauber et al., 2008). Thus, the proper quantification of the relative influence of soil type and plant species on plant associated bacterial community can only be achieved by sampling the same plant species in a gradient of soil types, while controlling for environmental conditions and meta-communities. Soils undergoing primary succession would provide such a perfect system.

In this study, we aim at exploring the importance of the selective force exerted by the plant in regulating the dynamics of bacterial communities around (rhizosphere) or inside (endosphere) the roots, as well as in the bulk soil, in different soil types, along a salt marsh primary succession gradient (Olff et al., 1997; Dini-Andreote et al., 2014, 2015). We chose *Limonium vulgare*, a typical perennial salt marsh plant, as our focus species because of its broad distribution along the chronosequence. The main hypotheses were based on the assumption that the selection by plants rather than soil would regulate the dynamics of the root-associated bacterial assembly along the chronosequence. Specifically, as a result of plant selection, we expect (i) phylogenetic diversity to decrease as we intensified the association with the plant—higher in bulk soil and lower in the endosphere—regardless of the soil successional stage. Moreover, we expect (ii) diversity to be constant within the rhizosphere and endosphere across different successional stages but variable in the bulk soil. In addition, we predict (iii) the structure of the plant associated bacterial communities to cluster according to the degree of connection with plant rather than soil successional stages. Within each community, however, we expected (iv) the distribution of the bacterial patterns to cluster according to three main successional—initial, middle and late stages—following the soil and vegetation development along the chronosequence (Schrama et al., 2012). Finally, considering the general principle that the plants function as “filters” of soil organisms, we expect (v) the bacterial communities in the endopshere to be less variable than those associated with rhizosphere and bulk soil.

## Materials and methods

### Study site and sample collection

The salt marsh chronosequence that we investigated is located on the island of Schiermonnikoog, the Netherlands (53°30′N, 6°10′E), and spans more than 100 years of primary succession (Olff et al., 1997). The succession starts from the east and develops to the west of the island. Permanent plots have been monitored at different successional stages during the last 20 years to verify the space-for-time replacement in this chronosequence (van Wijnen et al., 1997). Salt marsh age at each successional stage was estimated from topographic maps, aerial photographs, and the thickness of the sediment layer accumulated on top of the underlying sand layer (Olff et al., 1997; Schrama et al., 2012). For this study, samples were collected in 2014 at locations with successional ages of 5, 15, 35, 65, and 105 years. Three sampling plots (5 × 5 m) within each of the locations were established at the similar base elevation (vertical position relative to mean sea level at the initial elevation gradient on the bare sand flats). A base elevation of 1.16 ± 2.2 cm (mean ± SE) above Dutch Ordnance Level was used to select the sampling plots in this study. Different base elevations are exposed to different inundation regimes and, therefore, possess unique successional trajectories (Olff et al., 1997). As the salt marsh developed, clay sediments trapped by vegetation increased the elevation of the soil surface by ~16 cm over 100 years of succession (Schrama et al., 2012).

Sampling was performed twice (May and August) on all of the triplicate plots in the five stages to investigate the differences in season. As mentioned above, in order to quantify the relative influence of soil type and plant species on plant associated bacterial community, *L. vulgare* was selected as the focal plant in this study, as it is one of the dominant plant species along the chronosequence, and occurring throughout the succession, from the successional age 5 years onwards, peaking in abundance at 35 years (Schrama et al., 2012). Within each plot, four healthy-looking *L. vulgare* of similar sizes with attached soil adhering to the intact roots were collected as a composite sample using sterile spades and gloves. Therefore, thirty composite samples in total were collected (5 stages × 3 plots for each stage × 2 seasons). Each sample was placed in a sterile plastic bag, sealed and transported to the laboratory within 24 h. Spades were sterilized with 70% ethanol between different sampling plots and plants. From each composite sample we sampled bulk soil, rhizosphere, and endosphere (see below).

### Bulk (non-rhizosphere) and rhizosphere sample pretreatment

The plants were separated carefully from the adhering soil without damaging the roots by gentle shaking. All bulk (non-rhizosphere) soil samples were sieved (2 mm mesh size) and stored at −20°C for DNA extraction and 4°C for physicochemical measurements. Ten grams of roots with tightly adhering soil (rhizosphere soil) were transferred to an Erlenmeyer flask containing 180 mL of sterile sodium pyrophosphate (0.1%). After 30 min of shaking at 200 rpm at room temperature, 0.5 mL of the suspension with rhizosphere soil was used for DNA extraction or stored at −20°C.

### Endosphere sample pretreatment

Plant roots (about 8 g) were thoroughly washed with running tap water, trimmed to remove adhering soil and dead tissues, and surface sterilized by immersion into 1.5% NaClO solution (3 min), 70% ethanol (3 min) and sterile distilled water (3 × 3 min). Sterility checks were performed by tissue-blotting surface-sterilized plant samples on R2A plates, and checking the plates after 2–7 days incubation at 28°C. Samples without bacterial growth were considered successfully sterilized and used for further study. The surface-sterilized root parts (5 g) were sliced with a sterile scalpel and immersed into 15 mL NaCl solution (0.9%). After shaking incubation for 1 h at 28°C, the suspension with root pieces was shaken using a horizontal vortex instrument (4 × 1 min, 30 s in-between). Large plant and fungal cells were removed with 5-μm filters (Sessitsch et al., 2012), and the residual cells were pelleted by centrifuging (12,000 × g, 10 min) and then stored at −20°C for DNA extraction.

### Soil physicochemical parameters measurements

Soil samples stored at 4°C were used for measuring nitrate (N-${\text{NO}}_{3}^{-}$), ammonium (N-${\text{NH}}_{4}^{+}$), pH, and soil water content (SWC). The remaining soil samples (4°C) were dried at 40°C, ground with a grinding mill, and then used for measuring sodium (Na), magnesium (Mg), calcium (Ca), potassium (K), phosphate (P), and total nitrogen (TN). Soil physicochemical analyses were carried out in collaboration with the Department of Community and Conservation Ecology in the University of Groningen.

Soil N-${\text{NH}}_{4}^{+}$ and N-${\text{NO}}_{3}^{-}$ content was measured by extraction of 12.5 g soil with 30 mL KCl (1 M) overnight. After filtering the suspension, the extract was analyzed for N-${\text{NH}}_{4}^{+}$ and N-${\text{NO}}_{3}^{-}$ on a continuous flow auto analyzer (Skalar-40) using a colorimetric method (Keeney and Nelson, 1982). Soil exchangeable elements (Na, Mg, Ca, and K) were measured by extraction of 5 g soil with ammonium acetate (1 M) for 1 h (Knudsen et al., 1982; Lanyon and Heald, 1982). The filtrate was then analyzed on an atomic absorption spectrometer (AAS) set. Soil P was tested by extraction of 2.5 g soil with 5% HCl for 4 h. The filtered extract was then diluted with deionized water and a color developing reagent was added. The color intensity was measured at a wavelength of 420 nm on spectrophotometer after 1 h (Olsen and Sommers, 1982). The loss on ignition (LOI) method was applied for measuring soil organic matter (SOM) (Schulte and Hopkins, 1996). After soil samples had been measured for SWC at 105°C, they were placed in a muffle furnace at 550°C overnight, cooled down to room temperature in desiccators, and weighed. Soil texture data (sand:silt:clay % content) were referred to Dini-Andreote et al. (2014). We pooled the data of soil physicochemical parameters from both sampling periods, as the variation in soil environment between these two seasons (spring and summer) was small according to Dini-Andreote et al. (2014).

### Total DNA extraction and multitag pyrosequencing of partial 16S rRNA gene

A total of 30 bulk soil samples and 30 rhizosphere samples (five successional stages × two sampling times × three replicates) were subjected to total DNA extraction using 0.5 gram of soil and 0.5 mL of the suspension with rhizosphere soil, respectively, using the MoBio PowerSoil DNA extraction kit (MoBio Laboratories, Carlsbad, CA, USA). We followed the instruction manual, except for the addition of glass beads (diameter 0.1 mm, 0.25 g) to the MicroBead tube and three cycles of bead beating (mini-bead beater, BioSpec Products, USA) for 60 s. A total of 26 composite endophytic cell pellet samples (four stages × two sampling times × three replicates; 4 samples from the 5 year stage (including 3 samples from May and 1 sample from August) were excluded because of very small pellets) were subjected to total DNA extraction using the MoBio UltraClean Microbial DNA Isolation Kit (MoBio Laboratories, Carlsbad, CA, USA). We followed the instruction manual, except for heating the preparations at 65°C for 10 min with occasional bump vortexing for a few seconds every 2–3 min. Extracted DNA was further quantified using the PicoGreen dsDNA assay (Invitrogen, Carlsbad, CA, USA) within the wavelength range of 485–535 nm.

Twenty-five μL PCR reactions were performed using 0.25 μL 5U μL^−1^ FastStart High Fidelity (FSHF) Taq DNA Polymerase, 2.5 μL 10 × FSHF Reaction buffer without MgCl_2_, 2.3 μL 25 mM MgCl_2_ stock solution, 0.5 μL 10 mM PCR nucleotide mix, 0.25 μL 20 mg mL^−1^ bovine serum albumin (BSA) (Roche Diagnostics GmbH, Mannheim, Germany), and 0.5 μL each of 10 μM primer and 5 ng DNA template. The thermal cycler protocol was 95°C for 5 min, 30 cycles of 95°C for 40 s, 56°C for 45 s, 72°C for 40 s and a final 10-min extension at 72°C. The primer set 515f/1061r targeting regions V4–V6 of the 16S rRNA bacterial gene was used, which can provide sufficient resolution for the precise taxonomic classification of microbial sequences (Liu et al., 2007) and amplify 16S rRNA genes from a wide range of bacterial groups with few biases (Bates et al., 2011). For multiplexing, both primers were tagged using a unique short nucleotide sequence (10-bp) named “MID” for Roche GS-FLX 454 pyrosequencing, which act as barcodes to distinguish each sample and to detect external contaminants from sample DNA sequences (Parameswaran et al., 2007).

Pooled triplicate amplicons were run with 1% (w/v) agarose gel, and the gel containing the extract bands was excised and purified using the QIAquick Gel Extraction kit (QIAGEN GmbH, Hilden, Germany), and then quantified using PicoGreen dsDNA assay (Invitrogen, Carlsbad, CA, USA). Amplicons from all samples were pooled in equimolar concentrations into four composite samples containing 25, 25, 27, and 29 samples, respectively, and sequenced at Beckman Coulter Genomics (Danvers, MA, USA) on a Roche GS-FLX 454 automated pyrosequencer running Titanium chemistry.

### Sequence processing

Before analyses, raw sequencing data generated from the 454-sequencing runs were demultiplexed and processed using the Quantitative Insights into Microbial Ecology (QIIME) toolkit (Caporaso et al., 2010b). Briefly, the multiplex reads were assigned to samples based on their unique nucleotide barcodes with quality filtration using the default parameters, except that the range of the sequence lengths was set from 300 to 900. Quality filtered data was then denoised using Denoiser (Reeder and Knight, 2010). After denoising, the quality reads were binned into operational taxonomic units (OTUs) at 97% sequence similarity using UCLUST (Edgar, 2010), followed by picking a representative sequence for each OTU. Chimeric sequences were identified using ChimeraSlayer (Haas et al., 2011) and then removed. The representative sequence for each OTU was aligned referring to the Greengenes core set template (DeSantis et al., 2006) using PyNAST (Caporaso et al., 2010a), and then assigned to respective taxonomy identity with RDP classifier (Cole et al., 2005). The alignment was filtered to remove gaps in every sequence prior to the phylogenetic tree construction with FastTree (Price et al., 2009). For all OTU-based analyses, the original OTU table was rarified to a depth of 760 sequences per sample (the minimum number of sequences per sample) to minimize the sampling bias for analysis. Beta-diversity (weighted UniFrac distance in this study; Lozupone et al., 2006) and alpha-diversity metrics (the count of unique OTUs, Faith's phylogenetic diversity indices and Shannon diversity index) were also generated by QIIME. All sequencing data have been deposited in the MG-RAST database (http://metagenomics.anl.gov/).

### Data analyses

Significant differences in taxonomic relative abundance and alpha-diversity among different successional stages and different sources (bulk soil, rhizosphere, and endosphere) were identified using One-way analysis of variance (ANOVA), followed by Tukey HSD pairwise group comparisons in the R environment (http://www.r-project.org). To test the significance of the dissimilarity among different sources and different successional stages in the weighted unifrac distance matrix, we used permutational multivariate analysis of variance (PerMANOVA) (Anderson, 2001) in the vegan package (Oksanen et al., 2007). Constrained Analysis of Principal Coordinates (CAP) was applied on the weighted unifrac distance matrix by constraining the influence of sources and successional stages, in order to show the community clustering effects under the corresponding influence. The method investigates the results of a Principal Coordinates Analysis (function *cmdscale*) with linear discriminant analysis (lda), resulting in the best prediction of group identities of the sites (Anderson and Willis, 2003). The significance of the differences among different successional stages were tested by using the function *pairwise.perm.manova* within the RVAideMemoire package, to perform the pairwise comparisons using PerMANOVA on the weighted unifrac distance matrix. Different clusters separated by sources or by successional stages were visualized by using the function of *ordihull* in the vegan package. Changes in beta-diversity between two-adjacent succession stages along the chronosequence were calculated using the weighted UniFrac distance matrice. Graphs of alpha-diversity, taxonomic composition and variations of beta-diversity were constructed using Origin Pro 8.5 software.

To explain the partition of variation in the weighted-unifrac distances for bulk soil, rhizosphere, and endosphere, we used Constrained Analysis of Principal Coordinates (CAP) (Anderson and Willis, 2003) in the vegan package. Before being input into CAP analysis, soil physicochemical parameters were pre-filtered by using the *bioenv* function in the vegan package to select the best subset of environmental variables with maximum correlation with community dissimilarities (Clarke and Ainsworth, 1993). The pre-filtered parameters were then combined with soil texture indicators (Sand, Silt, and Clay%) as total soil factor. Venn diagrams were performed to shown the explained variation by soil factor, successional stages and seasonal changes.

## Results

### Site characteristics

A detailed description of the soil data is given in Table S1. In brief, compared to other successional stages, the 5-year stage showed the lowest level of nutrients (TN, SOC, ${\text{NO}}_{3}^{-}$, NH4^+^, P_2_O_5_, Ca, Mg, K), salinity (measured as the concentration of sodium, expressed in mg/kg dry soil), and soil water content (SWC). The soil pH, however, was highest at the 5-year stage (mean pH 8.51 ± 0.07) and decreased over succession (mean pH 7.51 ± 0.16 at the 105-year stage). With the development of the succession and sedimentation caused by the tidal regime, the soil physicochemical conditions and the level of silt and clay particles on the salt marsh with progressively increasing elevation were found to improve along the chronosequence, reaching a peak at the 65-year stage (except for nitrate levels). In addition, the salinity level also increased over time during succession, due to an accumulative effect.

### α-diversity measurements

**Figure 1:**
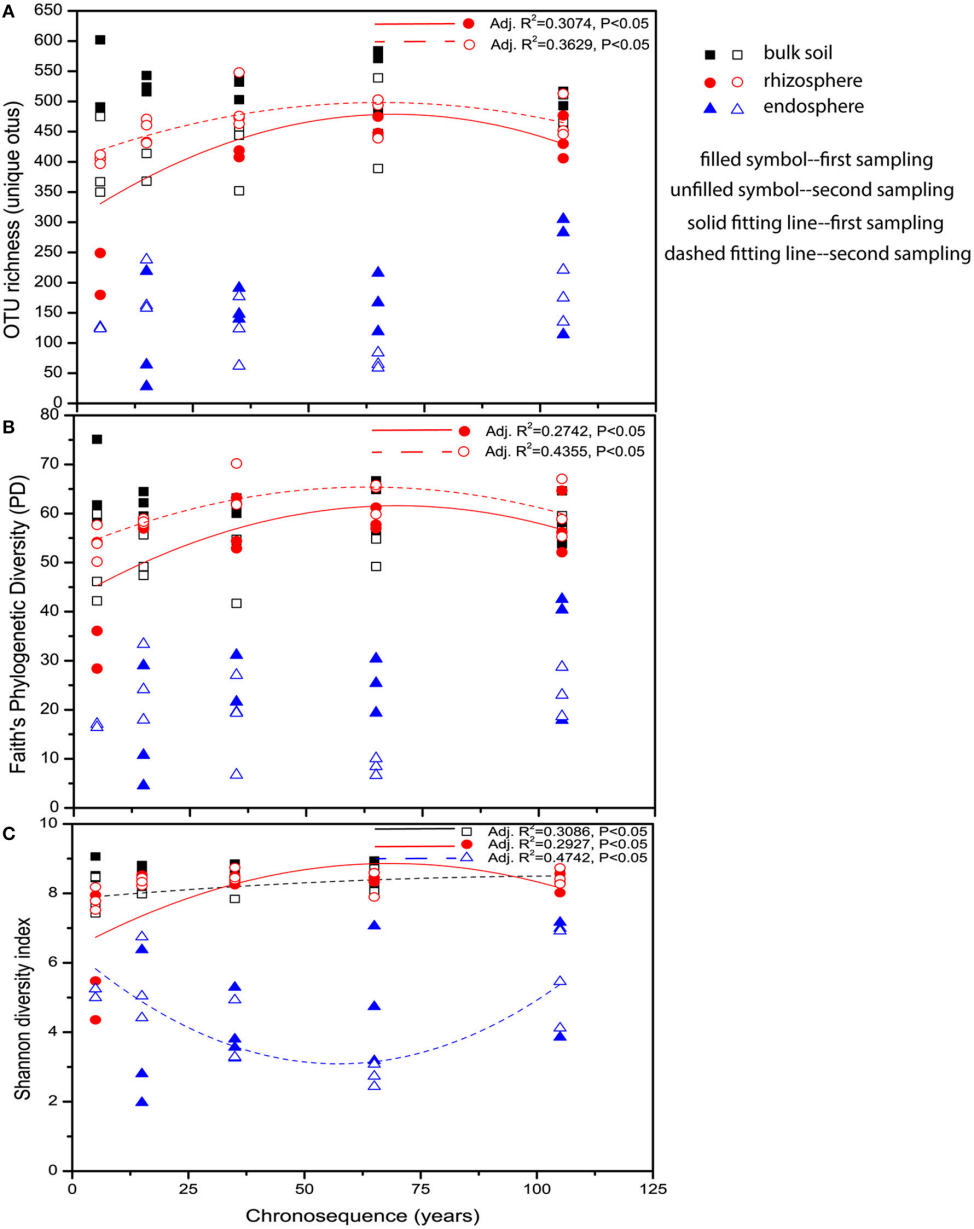
**Variation patterns of α-diversities of the bacterial communities associated with bulk soil, rhizosphere and endosphere along the chronosequence**. **(A)** OTU richness, **(B)** Faith's phylogenetic diversity (PD) and **(C)** Shannon diversity index. The polynomial models for each of the indices were performed using Origin Pro 8.5 software. Samples color coding: black, bulk soil; red, rhizosphere, and blue, endosphere.

To investigate the diversities of the bacterial communities associated with bulk and rhizosphere soil and endosphere along the chronosequence, the pyrosequencing data were rarefied to the depth of 760 reads per sample after being binned into 46,972 different OTUs, of which 48.76% of the sequences (22,905 OTUs in total) were singletons. Similar α-diversity values were shown for bulk soil and rhizosphere, which were significantly higher than those observed for endosphere (Figure 1), partially confirming our hypotheses that diversity should decrease as the association with the plant is intensified. Regarding the variation in diversity along the chronosequence, we observed no significant differences in Faith's phylogenetic diversity and OTU richness between stages nor patterns for endosphere and bulk soil communities, whereas for the rhizosphere soil both measures of diversity were significantly lower at the 5-year stage and peaked at the 65-year stage (Figures 1A,B). In terms of Shannon diversity, a similar hump-shaped pattern for the rhizosphere soil community from the first sampling and an increasing tendency for the bulk soil community from the second sampling were shown. For the endosphere, however, a reverse hump-shaped variation was found for the community from the second sampling, reaching the lowest point at the 65-year stage (Figure 1C). All these α-diversity results lead us to reject the prediction that diversity would be constant within the rhizosphere and endosphere but follow a successional pattern in the bulk soil. In fact, only rhizosphere-associated communities seem to corroborate the changes in the biotic and abiotic variables associated with the different successional stages.

### Phylogenetic β-diversity

**Figure 2:**
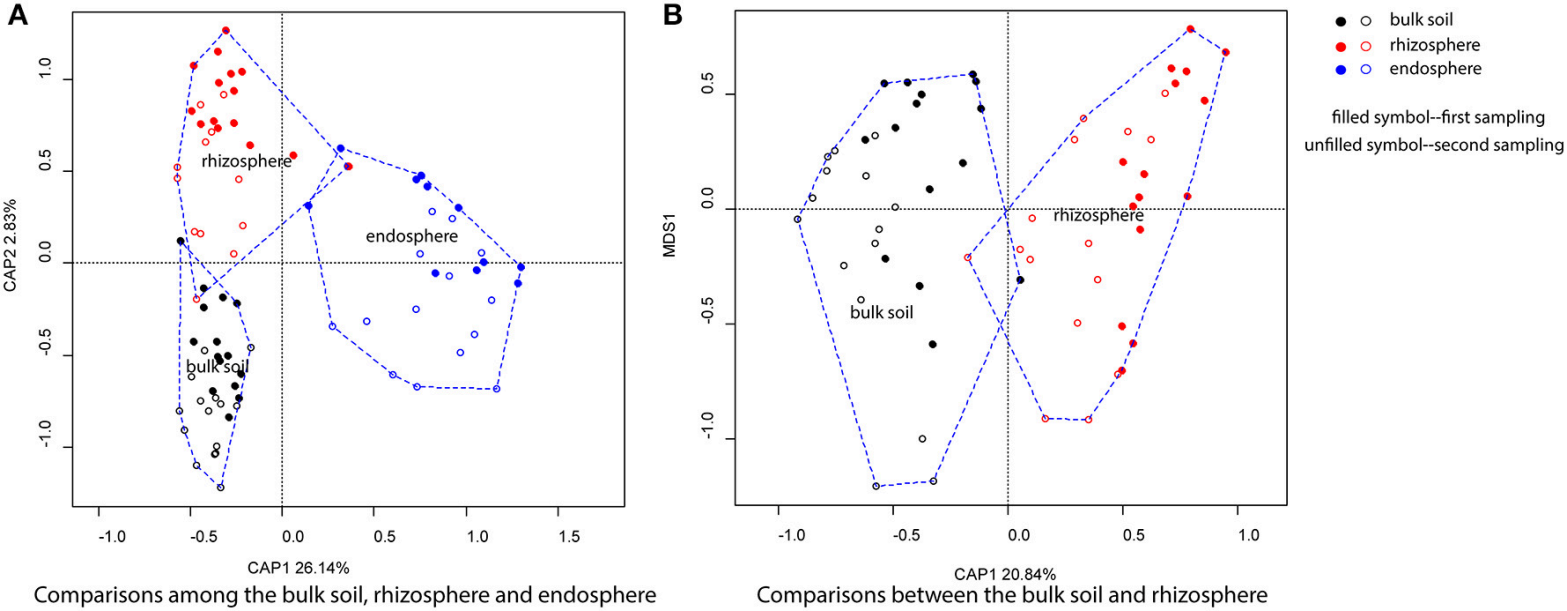
**Bacterial community structure of bulk soil, rhizosphere and endosphere**. **(A)** Comparisons among the bulk soil, rhizosphere, and endosphere, **(B)** Comparisons between the bulk soil and rhizosphere. Principal Constrained Analysis of Principal Coordinates (CAP) based on weighted-Unifrac distances was applied.

The clustering of bacterial communities from the bulk soil, rhizosphere and endosphere at different successional stages, performed with CAP analyses by using sources as the constraint. The first two constrained axes explained 28.97% of the total variation, with a significant effect of source (Pseudo-F = 17.192, *R*^2^ = 0.29, *P* < 0.001), successional stage (Pseudo-F = 3.136, *R*^2^ = 0.134, *P* < 0.001) and sampling time (Pseudo-F = 4.117, *R*^2^ = 0.047, *P* < 0.01; Figure 2A). Regarding the level of relationship with the plants, bacterial communities associated with the endosphere were significantly distinct from the bulk soil and rhizosphere. In order to further identify the community clustering effects between the bulk soil and rhizosphere, a similar analysis was performed but now excluding the endosphere samples (Figure 2B). The bulk soil and rhizosphere were significantly distinguished from each other (Pseudo-F = 3.85, *R*^2^ = 0.06, *P* < 0.001) with 20.84% of the total variation explained by CAP1, and there were a significant seasoning effect (Pseudo-F = 5.615, *R*^2^ = 0.088, *P* < 0.001) and a more important effect of successional stage (Pseudo-F = 5.40, *R*^2^ = 0.28, *P* < 0.001).

**Figure 3:**
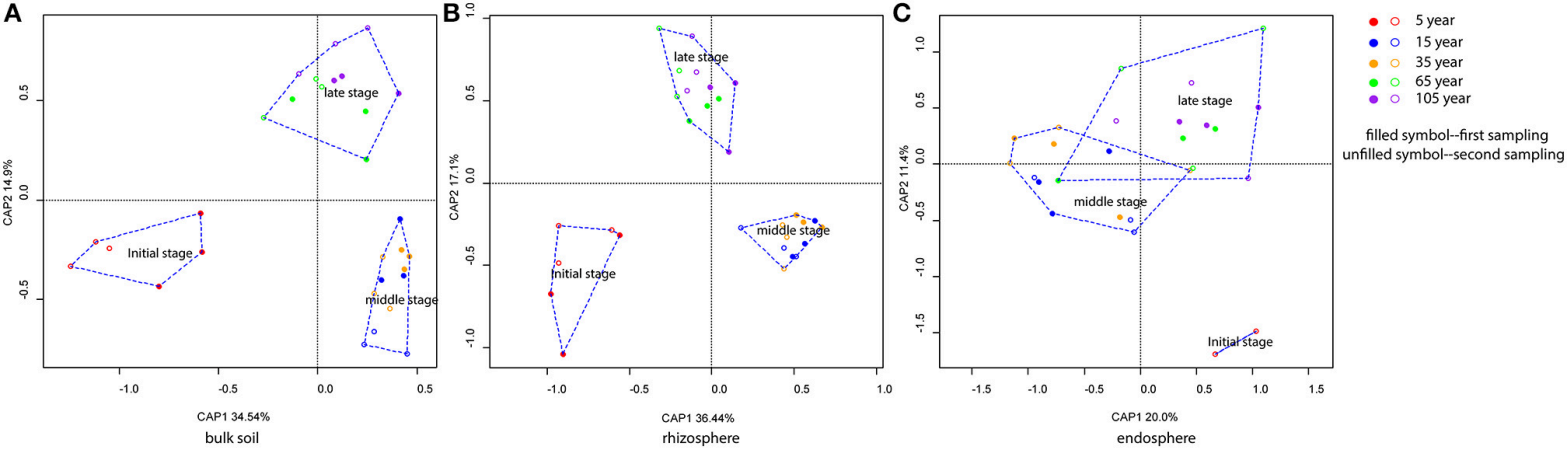
**Bacterial community structure of bulk soil, rhizosphere, and endosphere over the succession**. **(A)** Bulk soil, **(B)** Rhizosphere, and **(C)** Endosphere. Constrained Analysis of Principal Coordinates (CAP) based on weighted-Unifrac distances was applied. Samples color coding: red, 5 years; blue, 15 years; orange, 35 years; green, 65 years; and purple, 105 years. Filled symbols represent the samples from the first sampling time (May), and unfilled symbols represent the samples from the second sampling time (August).

**Figure 4:**
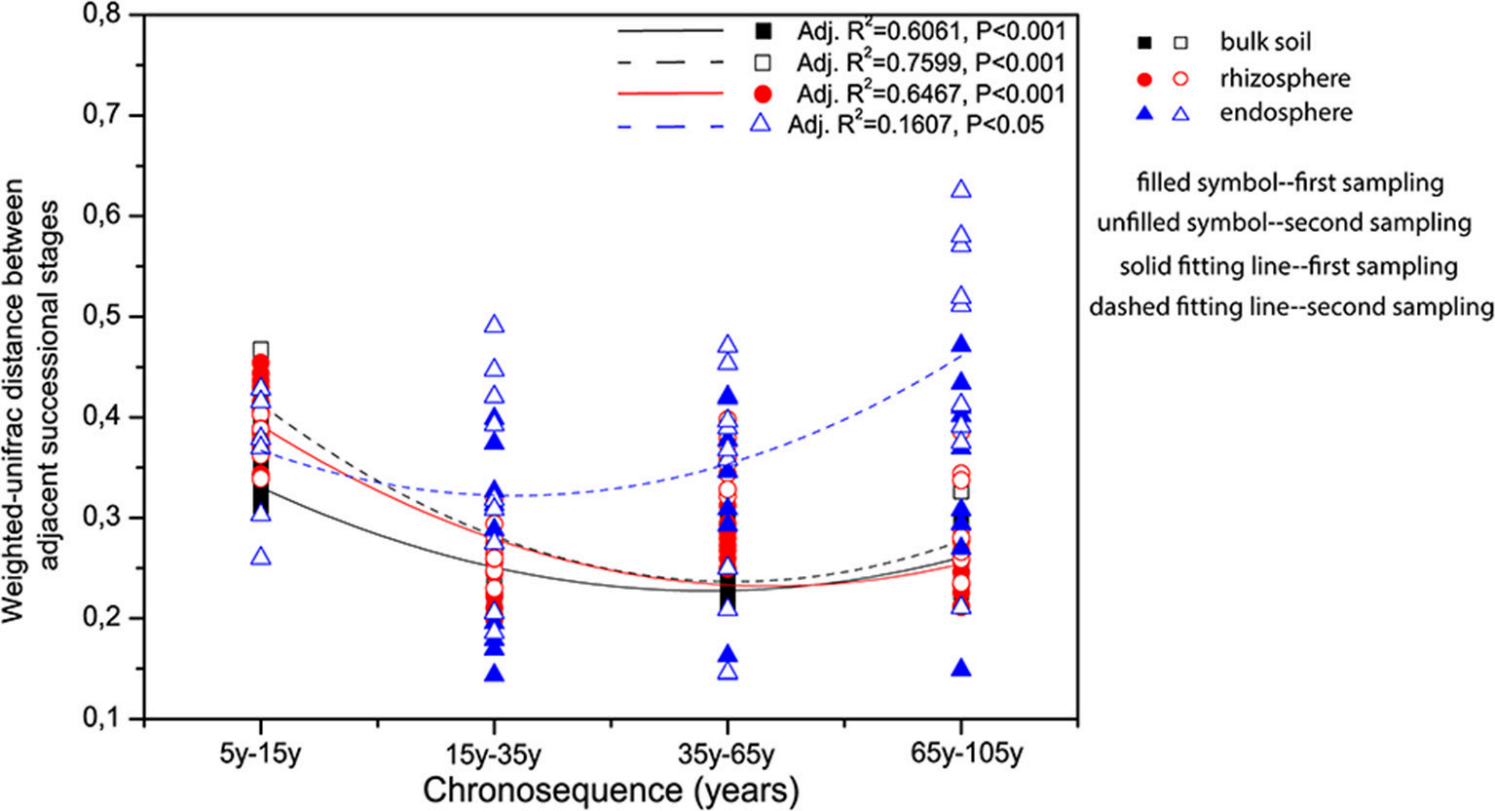
**Community dissimilarities along the chronosequence for the bulk soil, rhizosphere, and endosphere**. Variations in community dissimilarities were calculated with weighted-Unifrac distance between two adjacent successional stages. The polynomial models performed using Origin Pro 8.5 software. Samples color coding: black, bulk soil; red, rhizosphere, and blue, endosphere.

CAP analyses for bulk soil, rhizosphere, and endosphere individually revealed a clear clustering effect of each successional phase (initial, middle, late), by using successional stage as the constraint (Figure 3). Bulk soil and rhizosphere showed similar patterns—the community structures in the three successional phases were significantly different from each other (pairwise comparisons, *P* < 0.001) and reflecting the influence of successional phase as well as sampling time. Interestingly, the clustering effect within each phase became stronger after the initial stage, reaching the highest level at the middle stage (Figures 3A,B). On the contrary, endosphere-associated bacterial communities were highly dispersed when compared to the two other sources. Although significant differences were observed between middle and late phases (pairwise comparisons, *P* < 0.05), the difference level between each other was not as strong as that for bulk soil and rhizosphere. In addition, the dispersion level of the community structure within each successional phase was found to increase toward the late succession (Figure 3C). Indeed, by plotting the changes in community structure from one stage to the next, we could show that the turnover in community composition significantly decrease (*P* < 0.001) along the succession for bulk soil (from both samplings) and rhizosphere (from the first sampling) associated bacterial communities—indicating a more stable community structure as succession proceeded—whereas for the endosphere, the differences between communities in initial stages was lower than at late stages of succession (i.e., higher turnover; Figure 4).

**Figure 5:**
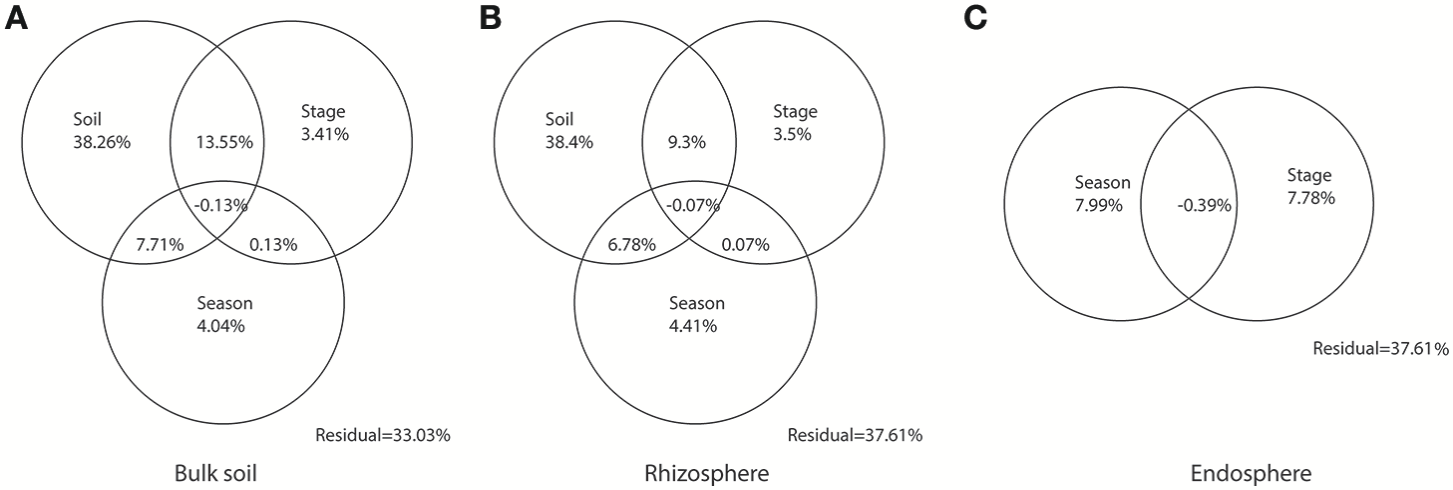
**Partitions of variation in the community composition of bulk soil, rhizosphere, and endosphere**. Venn graphs were performed according to the Constrained Analysis of Principal Coordinates (CAP) on the weighted-Unifrac distances of the bacterial communities within **(A)** Bulk soil, **(B)** Rhizosphere, and **(C)** Endosphere. Circles were not drawn to scale.

By partitioning the variation in the community dissimilarities of bulk soil, rhizosphere, and endosphere, we found that soil characteristic, including the selected subset of soil physicochemical parameters (pH, SWC, TN, and Na) and the soil texture indicators (sand, silt, and clay content), explained much higher partition of the variation in community dissimilarities for the microbiome associated with bulk soil (38.26%) and rhizosphere (38.4%), compared with successional stages and seasonal changes (Figure 5). Similarly, the successional stages and seasonal changes explained only small partitions of variation in the community dissimilarity in endosphere (7.78 and 7.99%, respectively).

All together, these results partially corroborate our hypothesis that bacterial community structure is determined by plant rather than successional stage. This was especially true for endophytes, where the effect of successional stages was completely absent. As expected, clustering according to successional stage was detected for bulk and rhizosphere bacterial communities. Intriguingly, endophytes experienced higher turnover in community composition at later stages of succession, contradicting our prediction that plant selection should lead to more similar communities.

### Bacterial community composition

#### Among bulk soil, rhizosphere, and endosphere

At the phylum level (Figure S1), Proteobacteria dominated the bulk soil, rhizosphere, and endosphere (51.6, 49.9 and 85.9%, respectively), followed by Bacteroidetes (18.2, 16.1, and 5.3%, respectively). The endosphere contained more Proteobacteria (*P* < 0.05) compared with the bulk soil and rhizosphere. The rhizosphere was found to accumulate significantly higher Chloroflexi and Planctomycetes (*P* < 0.05) than the bulk soil and endosphereat the middle stages (Figure S2).

Within the most predominant phylum, Proteobacteria, the classes Alpha- and Gammaproteobacteria were more abundant than Beta- and Deltaproteobacteria (Figure S2). Among the three sources, the endosphere showed the highest value of Gammaproteobacteria across the succession (*P* < 0.05, except for the 5-year stage). Among the six main genera of Proteobacteria (Figure S3), the endosphere showed the highest percentage of *Marinomonas* (especially for the 15-, 35-, and 65-year stages), which was the most abundant genus, belonging to the family Oceanospirillaceae.

#### Along the chronosequence

Choloflexi and Gemmatimonadetes progressively increased along the chronosquence within the bulk soil and rhizosphere (Figure S4). In the endosphere, a progressively increasing trend was also found for Firmicutes (*P* < 0.05). At the class level of the phyla Proteobacteria, Deltaproteobacteria increased significantly across succession in the bulk soil and rhizosphere (*P* < 0.001). Conversely, Alphaprotobacteria in the bulk soil and rhizosphere were found to show decreasing patterns (*P* < 0.05). For Gammaproteobacteria, hump-shaped and inverse-hump-shaped patterns were found in the bulk soil and rhizosphere, respectively, reaching the peak at the 15-year stage (32.05 ± 5.42%) and the bottom at the 35-year stage (21.82 ± 4.07%). Despite being the most dominant genus, no significant pattern in relative abundance was shown for *Marinomonas* along the chronosequence (Figure S5). In addition, large variability among replicated endosphere samples was found at each successional stage.

## Discussion

In this study we directly examined the relative contribution of soil and plant effect on the diversity and structure of bacterial communities varying in their degree of association with plants, by making use of a gradient of soil development in a salt marsh primary succession. We specifically investigated the drivers of root-associated bacterial communities related to the rhizosphere and endosphere of *L. vulgare*, a salt mash plant that is present along most of the successional gradient.

By providing a comprehensive overview of the phylogenetic diversity of root associated bacterial community along the salt marsh chronosequence, we showed that bacterial communities associated with bulk and rhizosphere soil were equally diverse whereas those associated with the endosphere were significantly less rich, which has also been reported recently (e.g., Saleem et al., 2015). Interestingly, phylogenetic diversity in the rhizosphere microbiome progressively increased, following the plant diversity patterns along the chronosequence (Schrama et al., 2012), as they both peak at the same successional stage. This result indicated the influence of abiotic and biotic variables in regulating bacterial diversity within rhizosphere. The increasing nutrient level in the soil and the higher plant richness could enrich the rhizosphere niches by providing more soil nutrients (De Ridder-Duine et al., 2005) and root exudates (Compant et al., 2010), therefore, possibly increasing the rhizosphere-driven selection on the surrounding soil microbes. Although, these results rejected our hypothesis that diversity would be constant within the rhizosphere, in retrospect they provide stronger support for the effect of plant in selecting the rhizosphere microbiome and emphasize the intricate interactions between plant and soil environment.

In opposition to the soil or rhizosphere microbiome, endophytic bacterial communities are often simple, encompassing up to hundreds of different bacterial types (Hardoim et al., 2008). Hence, our data confirms that plants can function as true “filters” of soil microorganisms, selecting a rare, phylogenetically less diverse fraction of those that are successful, competent endophytes (Sessitsch et al., 2002; Compant et al., 2005; Saleem et al., 2015). This result partially confirmed our hypothesis that diversity should decrease as the association with the plant is intensified, although this effect was not observed for rhizosphere communities. Moreover, the endophytic diversity was relatively constant across the succession (Figures 1A,B), confirming our prediction. Due to the intensified association with plant hosts and less biotic and abiotic stresses in the internal plant tissues (Hallmann et al., 1997; Rosenblueth and Martínez-Romero, 2006; Schulz et al., 2006), root inhabiting endophytes were barely influenced by the increasing nutrient level along the succession.

We next focused on understanding the root-associated bacterial community turnover across successional stages, by assessing of the shifts bacterial β-diversity among bulk soil, rhizosphere and endosphere. The distinctive community structure established in each source corroborated our prediction that the bacterial communities would cluster according to the degree of connection to the plant (Figure 2). Thus, bacterial composition is determined by the selection the plant exerts on rhizosphere and endosphere (Germida et al., 1998; Barriuso et al., 2005; Hartmann et al., 2009; Gottel et al., 2011). Importantly, differences between the source of microbiome outweighed differences in successional stage (proxy for soil characteristics) and sampling time.

The clustering patterns according to successional stage was shown for bacterial communities within bulk soil and rhizosphere when those were analyzed separately (Figures 3A,B), confirming, for those two communities, our hypothesis that the distribution of the bacterial patterns would cluster according to three main successional phases. Specifically, we observed a unique cluster at the initial successional phase for soil and rhizosphere, which could be explained by the combination effects of the significantly lower nutrient level and higher flooding frequency (Table S1, Schrama et al., 2012; Dini-Andreote et al., 2014, 2015). For the other stages along the chronosequence, apparent clustering was shown for the middle and late successional phases, reflecting the accretion of nutrients in the soil, as well as clay and salt accumulation. In addition, the clustering effect within each phase became stronger over the succession indicating a lower turnover in community structure for bulk and rhizosphere soil towards the end of the succession (Figure 4). These data are consistent with successional dynamics of macro organisms, where the buffering effects of soil as well as plants becomes increasingly more dominant following the development of succession, leading to reduced community turnover and more stabilized community structure (Walker and del Moral, 2003; Dini-Andreote et al., 2014). Thus, for bulk and rhizosphere soil, we could conclude that as the variability of nutrients as well as immigration (mainly derive from marine associated microbial input, through flooding regimes) decreases as the succession precedes, the community structure becomes more similar. By partitioning the variation in the community dissimilarities, we found the changes in soil environmental conditions along the chronosequenceto drive the community turnover for bulk and rhizosphere soils (Figures 5A,B).

The endophytic bacterial communities, however, showed much higher dispersion level within each successional phase (Figure 3C). In addition to this, the increasing variation of community dissimilarity (Figure 4) contradicted our expectation that the endophytic bacterial communities would be less variable than those associated with rhizosphere and bulk soil. Although, plants did select for different microbiomes, which were less diverse and differently structured than those from the other sources (bulk and rhizosphere soil), thus acting as “filters” of the soil microbes (Sessitsch et al., 2002), this influence was barely linked to succesional stage nor soil type nor was this influence stable over time. Therefore, we suggest that except for the initial plant-selective force exerted on the endophytic community, other key factors also modulate the community structure in the internal root tissues, such as plant age (Zinniel et al., 2002; Kuklinsky-Sobral et al., 2004). Although, we looked for plants of similar sizes when sampling, we cannot reject the possibility that plants differ in age, which would be especially relevant in the later stages of succession. Moreover, we could speculate that the peak of *L. vulgare* abundance, which takes place at the intermediate stage of succession, indicates the most appropriate successional stage for this species. In that case, plants could be experiencing higher competition for nutrients and more demanding conditions due to the increase in salt concentration, consequently influencing the plant control over their microbiome, especially for endophytes. Although, we cannot pinpoint the specific factor leading to the higher turnover in endophytic communities at the end of the succession, the opposite trend observed for the communities associated with bulk soil and rhizosphere makes the selection pressure exerted by plants even more spectacular.

Finally, by delving into the taxonomic composition of root-associated bacterial assemblages along environmental gradients of the succession, we could detect a few patterns. For instance, Chlorofexi, Plantomycetes, and deltaproteobacteria increased in relative abundance in the rhizosphere of *L. vulgare* over the succession, whereas Gammaproteobacteria decreased, mostly due to a decrease in the genus *Pseudomonas*. The communities associated with endophytes were largely enriched in Proteobacteria, which was the predominant phylum across all samples. Within this phylum, the significant accumulation of the genus *Marinomonas* within the endosphere corroborated once more that the plant selective force plays a substantial role in regulating the endophytic bacterial community composition (Figure S3). The genus *Marinomonas*has been found among many different marine environments (Sanchez-Amat and Solano, 2005), in association with species associated with marine ecosystems such as seaweed (Singh et al., 2011) and salt marsh grass (Andrykovitch and Marx, 1988). The dominance of this group, which reached more than 70% of relative abundance of the total families in some of our samples, indicated their role as competent endophytes. The above studies on the *Marinomonas* suggest it could exert beneficial traits on the plant hosts, therefore leading to its dominance in endosphere. Contrary to *Marinomonas*, the genus *Pseudomonas* and *Cellvibrio*, could be considered as opportunistic endophytes as their relative abundances peaked only at the early stage of succession.

Overall, this study offers for the first time an overview of the variation of root-associated bacterial composition along a salt marsh chronosequence. Importantly, by focusing on non-domesticated plants in a natural ecosystem, we could show that the importance of soil in driving bacterial community composition is dependent on the plant compartments, being influential only in the rhizosphere but not on the endosphere. The distinctive dominant bacterial composition within endosphere validates the existence of the plant selective force on the root-associated microbes, resulting in the distinctive taxonomy composition and diversity in the rhizosphere and the internal root tissues.

### Conflict of interest statement

The authors declare that the research was conducted in the absence of any commercial or financial relationships that could be construed as a potential conflict of interest.
