# Supplementary material for: Distribution of Root-Associated Bacterial Communities Along a Salt-Marsh Primary Succession
Source: Front Plant Sci. 2016 Jan 5;6:1188. doi: 10.3389/fpls.2015.01188 (PMC4700203; doi:10.3389/fpls.2015.01188)
Supplement: Supplementary file 1 [file Table1.DOCX]

Table S1. Location and soil physicochemical parameters measured at the sampling sites along the salt marsh chronosequence on the island of Schiermonnikoog, the Netherlands. The soil physicochemical parameters were calculated by combining the results from two samplings taken in 2014.

|  | | 5 year | 15 year | 35 year | 65 year | 105 year |
| --- | --- | --- | --- | --- | --- | --- |
| Site Location  Latitude | | | | | | |
| Plots A | | 53°30’18’’N | 53°30’7’’N | 53°29’59’’N | 53°29’35’’N | 53°28’56’’N |
| Plots B | | 53°30’18’’N | 53°30’7’’N | 53°29’59’’N | 53°29’37’’N | 53°28’56’’N |
| Plots C | | 53°30’18’’N | 53°30’7’’N | 53°29’59’’N | 53°29’37’’N | 53°28’57’’N |
| Longtitude | | | | | | |
| Plots A | | 6°19’51’’E | 6°19’54’’E | 6°18’57’’E | 6°16’20’’E | 6°14’3’’E |
| Plots B | | 6°19’52’’E | 6°19’55’’E | 6°18’59’’E | 6°16’21’’E | 6°14’2’’E |
| Plots C | | 6°19’54’’E | 6°19’56’’E | 6°18’60’’E | 6°16’19’’E | 6°14’1’’E |
| Measured Parameters | | | | | | |
| pH | | 8.51±0.07 | 7.74±0.10 | 7.57±0.08 | 7.72±0.11 | 7.51±0.16 |
| soil water content (%) | | 6.64±2.04 | 40.24±4.81 | 46.32±6.56 | 63.83±0.92 | 57.53±4.96 |
| Soil organic carbon (%) | | 0.51±0.24 | 5.28±1.46 | 7.04±1.74 | 13.22±0.02 | 11.23±0.36 |
| Total nitrogen (%) | | 0.01±0.00 | 0.28±0.06 | 0.41±0.13 | 0.92±0.03 | 0.79±0.03 |
| Nitrate (mg/kg dry soil) | | 9.94±2.13 | 13.83±4.06 | 14.21±8.60 | 19.33±24.18 | 23.82±25.45 |
| Ammonium (mg/kg dry soil) | | 1.06±0.44 | 12.25±7.41 | 21.58±16.68 | 38.38±19.80 | 29.70±10.68 |
| Ca (mg/kg dry soil) | | 2096.16±107.81 | 2972.63±459.00 | 3676.96±166.78 | 3228.29±199.22 | 3154.78±607.00 |
| Mg (mg/kg dry soil) | | 75.71±15.71 | 967.49±363.97 | 1991.63±285.34 | 3134.24±162.20 | 2536.61±228.13 |
| K (mg/kg dry soil) | | 60.14±18.69 | 535.06±247.71 | 1143.26±213.16 | 1778.08±72.57 | 1557.98±176.68 |
| Na (mg/kg dry soil) | | 150.82±70.71 | 3851.51±1256.95 | 7788.34±426.92 | 7144.92±1144.14 | 7268.55±1230.72 |
| Available phosphate (P_2_O_5_; mg/kg dry soil) | | 284.08±3.04 | 810.69±37.99 | 1308.58±26.16 | 2226.62±15.63 | 2116.18±50.59 |
| Soil Texture | | | | | | |
| Sand (%) | 2.96±0.00 | | ⎯ | 2.73±0.01 | 2.29±0.02 | 2.13±0.02 |
| Silt (%) | 1.48±0.13 | | ⎯ | 2.35±0.01 | 2.64±0.02 | 2.69±0.01 |
| Clay(%) | 1.71±0.00 | | ⎯ | 2.37±0.02 | 2.56±0.01 | 2.58±0.00 |

Soil type data was not tested in the 15-year stage.
