# Supplementary material for: Distribution of Root-Associated Bacterial Communities Along a Salt-Marsh Primary Succession
Source: Front Plant Sci. 2016 Jan 5;6:1188. doi: 10.3389/fpls.2015.01188 (PMC4700203; doi:10.3389/fpls.2015.01188)
Supplement: Supplementary file 2 [file Presentation1.PPTX]

## Slide 1
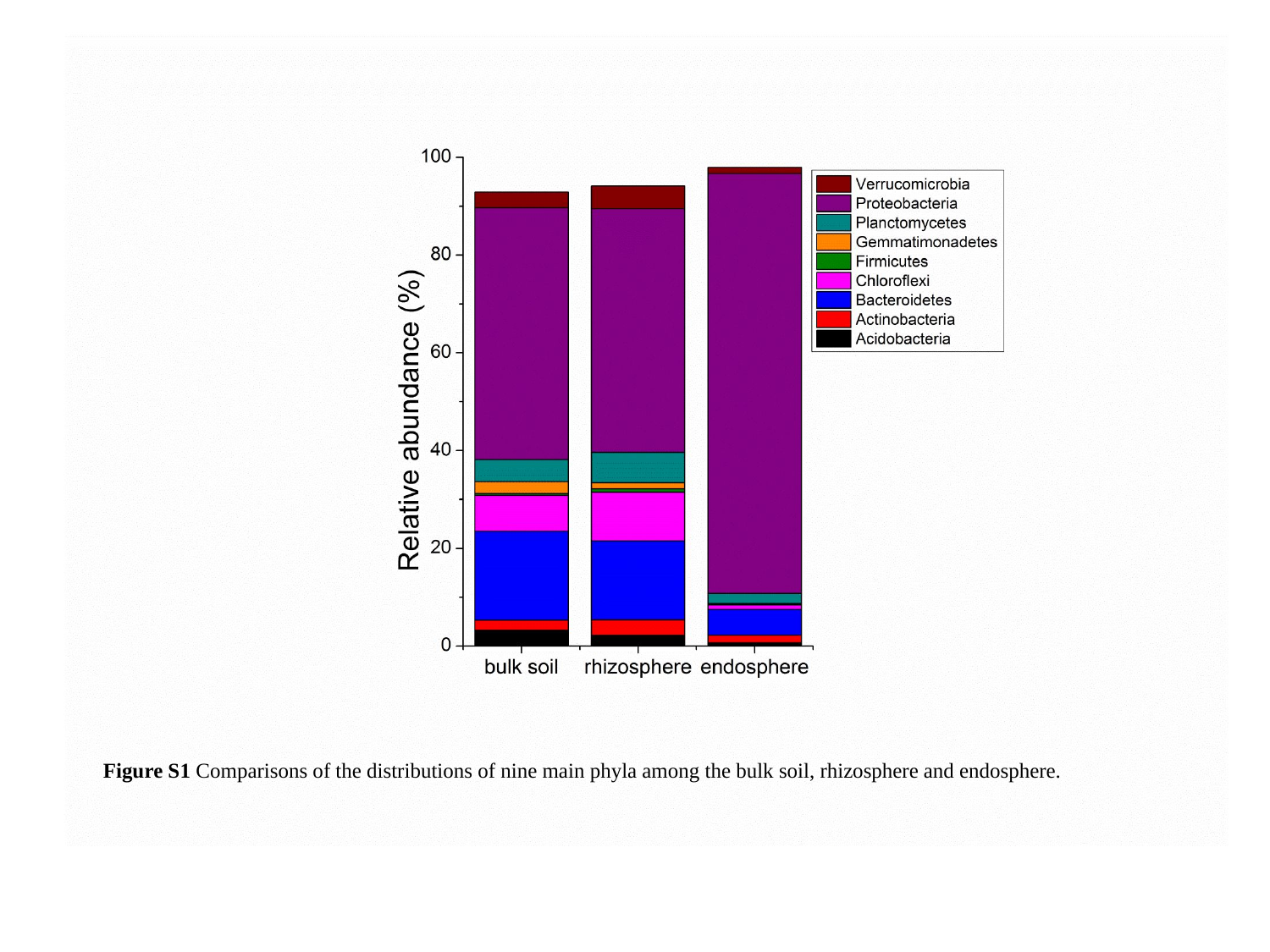

Figure S1 Comparisons of the distributions of nine main phyla among the bulk soil, rhizosphere and endosphere.

## Slide 2
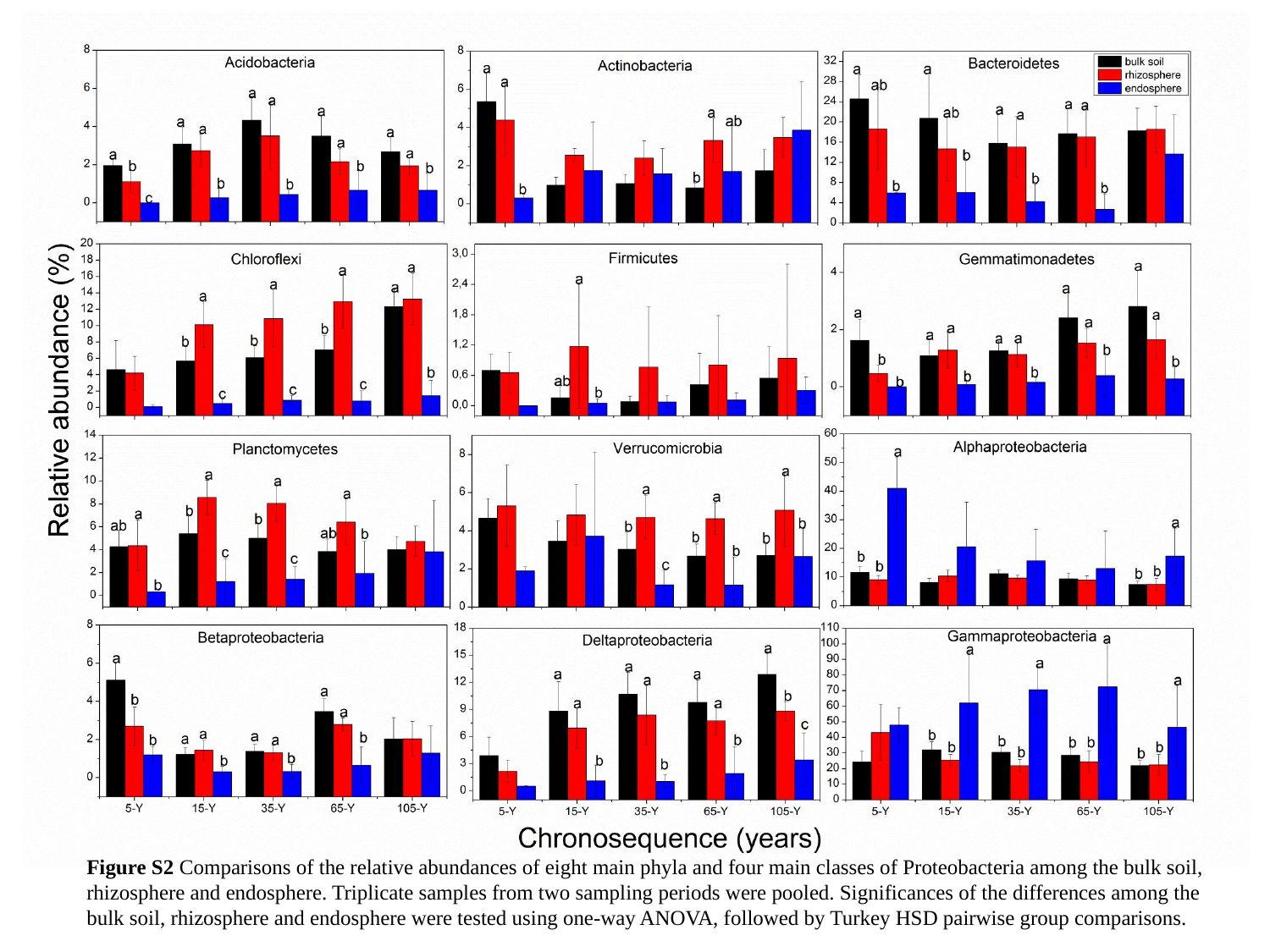

Figure S2 Comparisons of the relative abundances of eight main phyla and four main classes of Proteobacteria among the bulk soil, rhizosphere and endosphere. Triplicate samples from two sampling periods were pooled. Significances of the differences among the bulk soil, rhizosphere and endosphere were tested using one-way ANOVA, followed by Turkey HSD pairwise group comparisons.

## Slide 3
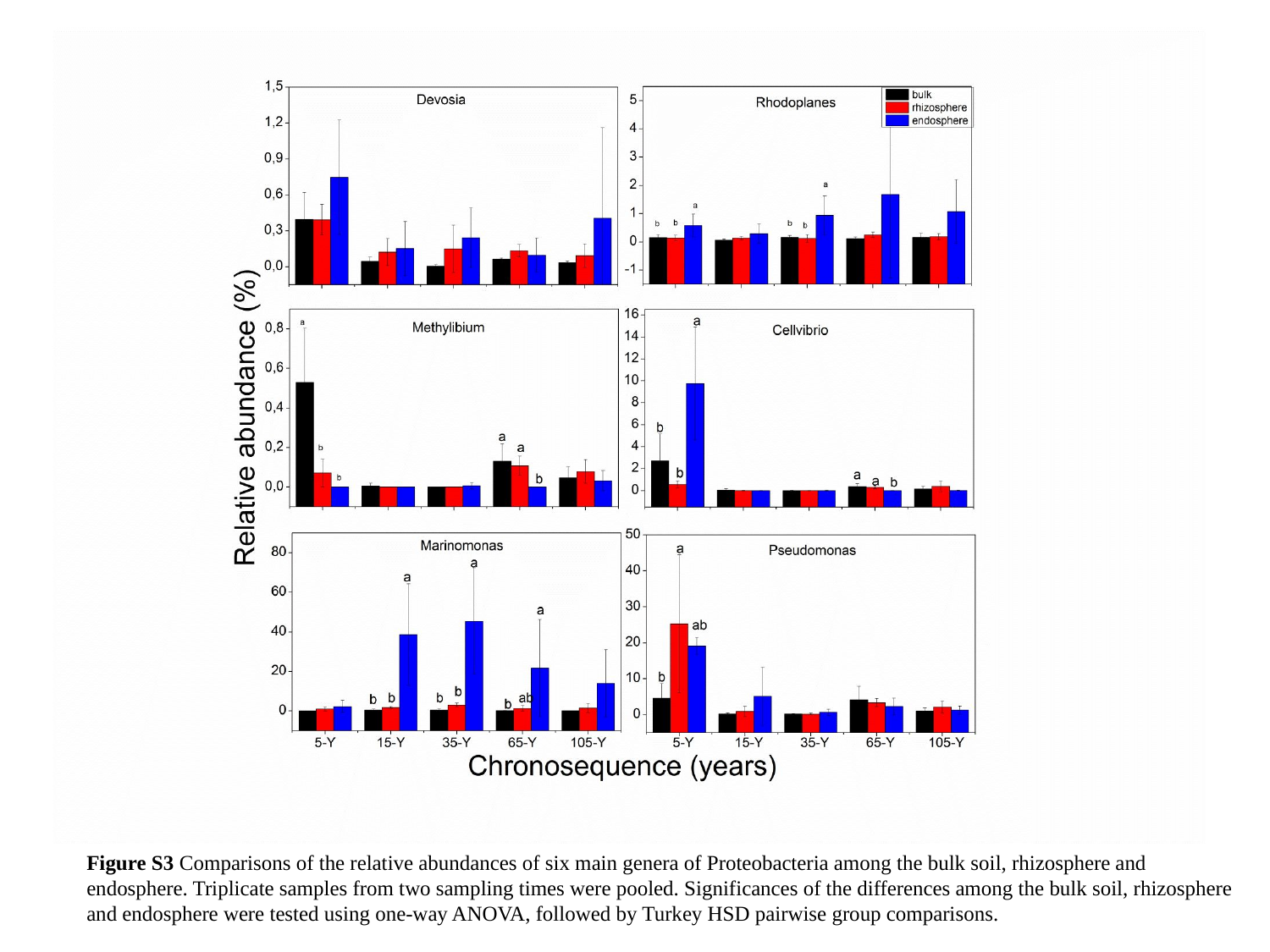

Figure S3 Comparisons of the relative abundances of six main genera of Proteobacteria among the bulk soil, rhizosphere and endosphere. Triplicate samples from two sampling times were pooled. Significances of the differences among the bulk soil, rhizosphere and endosphere were tested using one-way ANOVA, followed by Turkey HSD pairwise group comparisons.

## Slide 4
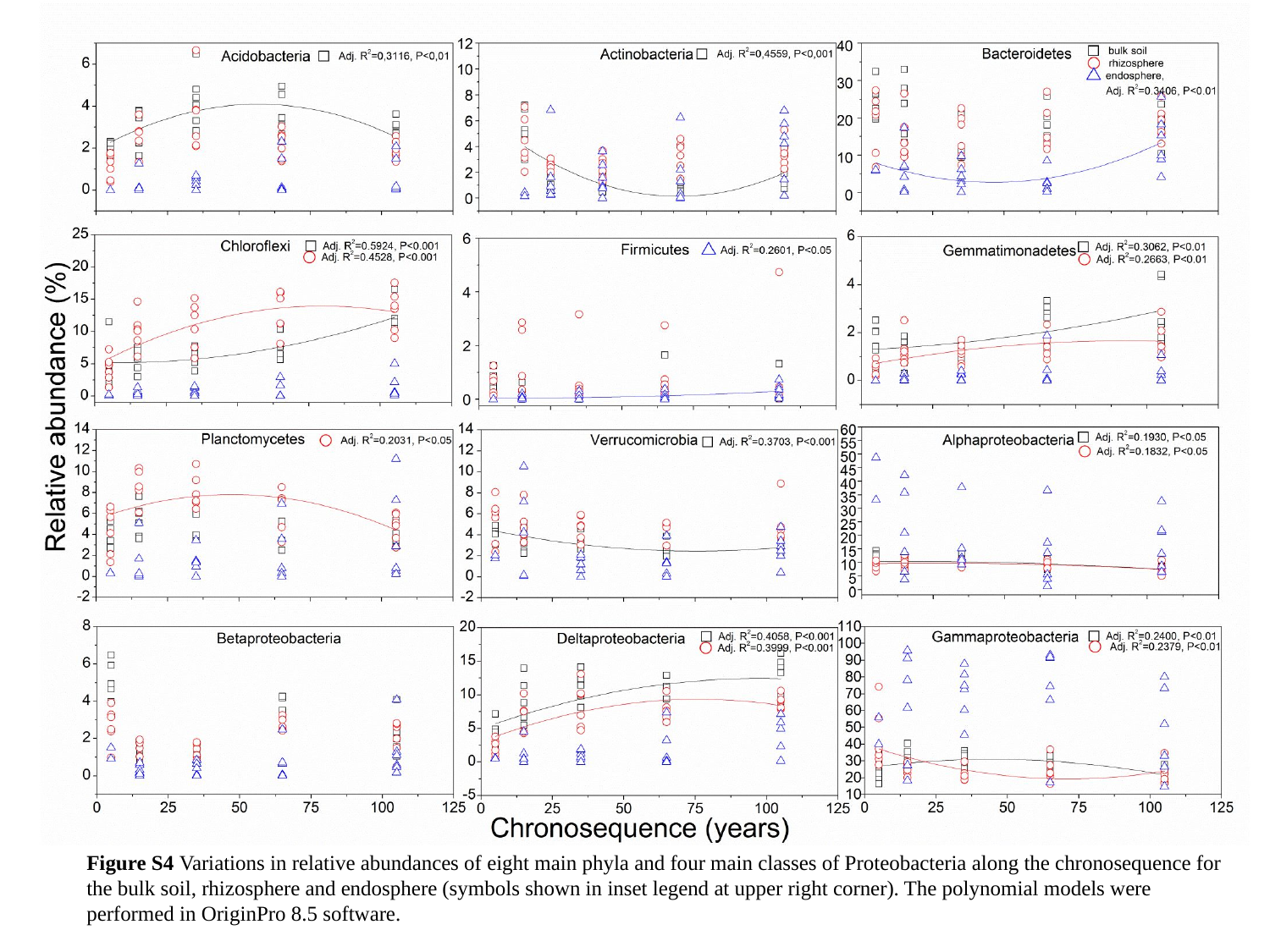

Figure S4 Variations in relative abundances of eight main phyla and four main classes of Proteobacteria along the chronosequence for the bulk soil, rhizosphere and endosphere (symbols shown in inset legend at upper right corner). The polynomial models were performed in OriginPro 8.5 software.

## Slide 5
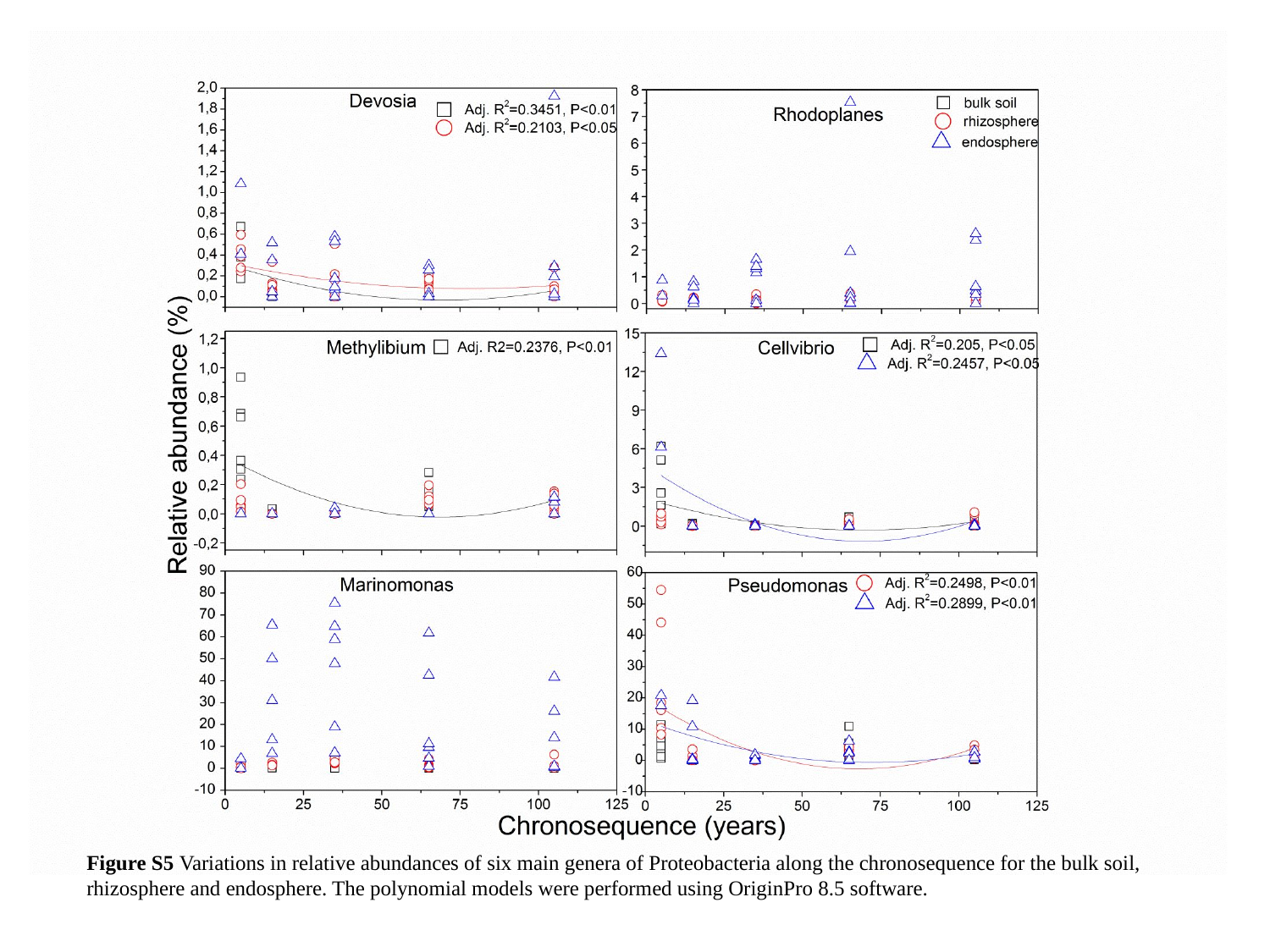

Figure S5 Variations in relative abundances of six main genera of Proteobacteria along the chronosequence for the bulk soil, rhizosphere and endosphere. The polynomial models were performed using OriginPro 8.5 software.
